# Home-based HIV testing: Using different strategies among transgender women in Argentina

**DOI:** 10.1371/journal.pone.0230429

**Published:** 2020-03-19

**Authors:** Claudia E. Frola, Virginia Zalazar, Nadir Cardozo, María L. Vázquez, Inés Arístegui, Mar Lucas, Ana Gun, Pedro Cahn, Omar Sued

**Affiliations:** 1 Division of Infectious Diseases, Juan A. Fernández Hospital, Buenos Aires, Argentina; 2 Clinical Research Department, Fundación Huésped, Buenos Aires, Argentina; 3 Association of Transvestites, Transsexuals, and Transgenders of Argentina (A.T.T.T.A.), Buenos Aires, Argentina; 4 Universidad de Palermo, Buenos Aires, Argentina; Ohio State University, UNITED STATES

## Abstract

**Background:**

In Argentina, HIV prevalence among transgender women (TGW) has been reported at 34%. The stigma is one of the most important factors limiting their access to healthcare services. The aims of this study were to compare different HIV testing methodologies, to determine the factors associated with HIV diagnosis and to determine the feasibility of a home-based HIV testing service for TGW.

**Methods:**

A multidisciplinary team performed home-based HIV testing interventions in four cities of Argentina. Participants self-identified as TGW, older than 14 years and with a negative or unknown HIV status. Blood samples were screened by two rapid tests (RT), one based on antibodies (Determine^™^ HIV-1/2) and the other on antigen and antibodies (Determine^™^ HIV-1/2 Combo), and the subsequent blood processing via 4^th^ generation ELISA (VIDAS HIV DUO). All reactive samples were confirmed with a viral load (VL). We compared the results of both RT with the ELISA. Samples were pooled in groups of 6 and a VL (Abbott Real Time) performed to identify acute HIV infections. Factors associated with HIV infection were evaluated with multivariate logistic regression analysis.

**Results:**

A total of 260 TGW were tested, 51 tested positive (HIV prevalence 19.6%). There were no discordant results between both RTs nor between RTs and 4^th^ generation ELISA, therefore the correlation was 100%. The VL identified 2 additional positive samples. The final analytic sample for positive cases consisted of 53 TGW. In the multivariate analysis, factors associated with a positive HIV result were *history of other sexually transmitted infections (STIs)* and *not being previously tested for HIV*. TGW tested for the first time were at 4 times greater risk of being HIV positive compared to those that were tested previously.

**Conclusions:**

A multidisciplinary home-based HIV testing service among TGW is feasible and effective to detect cases of HIV infection. The testing algorithm should start with an RT followed by molecular diagnosis. The history of STIs and never having been tested for HIV were the factors associated with HIV-positive results and should determine efforts to reach this population. Home-based testing reaches individuals that were not tested before and who have more risk of acquiring HIV.

## Introduction

Worldwide, transgender women (TGW) have an extremely high HIV prevalence compared to other adults, with almost 50 times higher odds of infection [1]. In Argentina, the burden of HIV infection among TGW has been reported as high as 34%, compared to 0.4% in the general population [2,3]. In addition to biological factors, structural components increase the risks for HIV infection and other sexually transmitted infections (STIs) in TGW. In most cases, social exclusion begins at an early age within their families and continues in the educational setting, limiting their later access to jobs and leading many TGW to engage in sex work. This scenario helps to understand the high rates of HIV among this population [4,5].

The implementation of a series of legal changes in Argentina in the last decade, such as the Comprehensive Sexual Education National Law [6], the Equal Marriage Law [7], and the Gender Identity Law [8], have promoted a favorable environment toward the full realization of LGBT (lesbian, gay, bisexual, and transgender) rights. In particular, the Gender Identity Law guarantees the legal recognition of self-defined gender identity and endorses the access to gender affirmation procedures [9]. In spite of these legal efforts, the inequities and social vulnerability among the TGW community still persist.

Due to the discrimination TGW encounter in their families, and the consequent marginalization and poverty, the majority of the TGW in the Buenos Aires metropolitan area live in shared rooming houses [10]. Moreover, this population also faces socio-structural inequalities when it comes to health services, stigma being one of the most important factors limiting their access to healthcare services [11]. Therefore, many TGW postpone medical consultation and only show up with extremely serious health conditions [12]. The aforementioned situations explain the low rate of HIV testing in TGW [13], and highlight the need to explore new testing strategies for this population. Outreach programs, where health services go out into the community for HIV/STIs screening, have proven to be an effective service in reaching at-risk populations, including sex workers [14]. Home-based testing involves door-to-door implementation of rapid testing (RT) done by professional and/or community health workers. Compared to health facility-based testing, this strategy is highly acceptable, as reduces HIV-related stigma in the health services [15]. Some strategies focused on improving healthcare access among TGW have been previously implemented in Latin America. In order to diminish stigma and discrimination by healthcare providers, some of the strategies successfully applied and welcomed by the community were the incorporation of TGW peer navigators in health services as well as mobile HIV and STI testing [16,17]. However, there is a lack of home-based HIV strategies applied in the transgender population in the Latin American context.

In order to achieve the UNAIDS global 90-90-90 HIV goals, it is necessary to focus on strategies that target key populations with the highest prevalence, incidence, and risk of transmission [18]. The high incidence of HIV in TGW and the barriers to accessing medical care require innovative strategies for earlier detection, ideally, with the capability to detect acute HIV infection (AHI); a period that extends from the acquisition of the virus to the development of the complete humoral immune response and that is associated with an extremely higher risk of onward transmission [19]. Therefore, early treatment can result in significant benefits for individuals as it can prevent immunological damage and reduce the size of the viral reservoir [20,21,22]. By definition, during this phase, the presence of the virus can be detected with HIV plasma ribonucleic acid (RNA) tests from the 7^th^ day onwards, with p24 antigen (Ag) at 10 days, and with tests based on antibodies (Ab) about 28 days after infection [23,24]. The use of tests with the capability to detect both Ab and p24 reduced the window period, and recently RTs were demonstrated to be as effective as 4^th^ generation ELISA [25]. However, as molecular techniques are more sensitive but also expensive, pooled specimens were proposed as a way to make RNA amplification tests cost-effective [26].

### Objective

Thus, the aims of this study were: a) to compare different HIV testing methodologies (RT based on Ab, RT based on Ag and Ab, 4^th^ generation ELISA and pooled viral load), b) to determine the factors associated with and HIV-positive diagnosis, and c) to determine the feasibility of a multidisciplinary home-based HIV diagnosis intervention for TGW.

## Methods

### Participants

Participants were self-identified as TGW, older than 14 years and with unknown HIV status or a previous negative test (≥3 months).

### Study procedures

In the *preparatory phase*, we formed a multidisciplinary team (infectious disease physicians, psychologists, laboratory technicians, and peer health promoters) and designed a home-based intervention to facilitate HIV diagnosis based on the results of a previous study aimed at identifying barriers to healthcare access and the feasibility of different interventions among TGW [27]. In this previous study, some rooming houses were selected for home-based interventions and later, we expanded the search to community-based organizations and other venues where TGW congregate, with the assistance of TGW health promoters. Additionally, we mapped corresponding health centers to each venue in order to establish facilitated referral for HIV-positive cases identified.

In the *intervention phase*, from November 2015 to August 2017, the multidisciplinary team performed 28 home-based HIV diagnosis interventions in four cities (Buenos Aires city, Greater Buenos Aires, Santiago del Estero and Salta) of Argentina. After signing informed consent forms, participants were counseled in risk reduction for sexual exposure and condoms were provided. All cases were registered in a confidential manner, using a code consisting of initials and date of birth.

**Fig 1:**
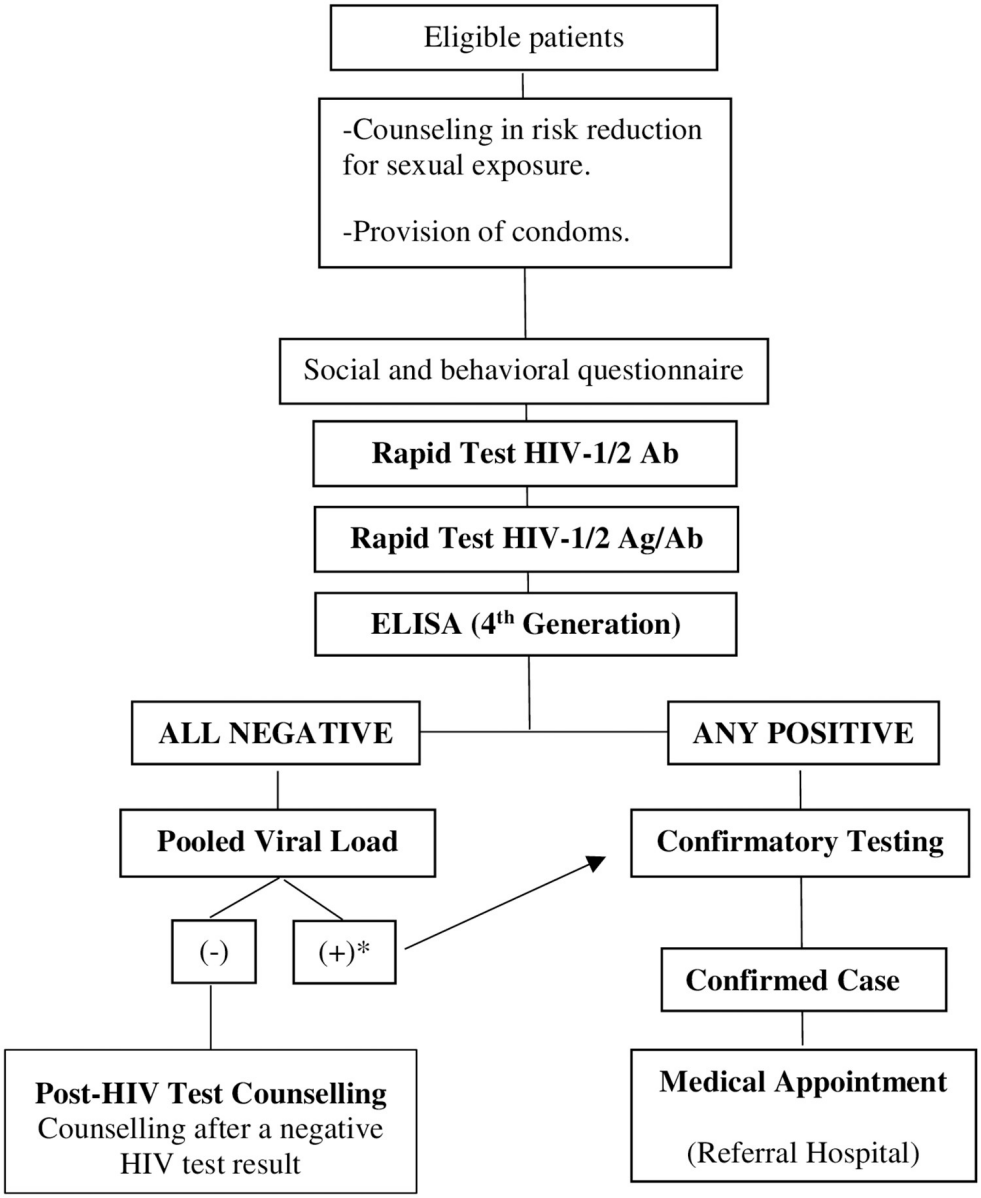
Flow chart of the HIV testing algorithm. *All the samples were analyzed individually by viral load to identify the positive sample(s). Ab, antibodies. Ag, antigen.

Additional information was collected through a social and behavioral questionnaire designed ad-hoc. Questions included in this study were: age; gender (female, male, trans, other- in case of TGW, both female and trans were checked); nationality; “have you ever had an HIV test?” (no, first time; yes, it was positive; yes, it was negative; yes, I didn’t return for the result; NR/DK); “did you use a condom in your last sexual encounter?” (yes, no, NR/DK), “have you ever had a sexual partner with HIV?” (yes, no, NR/DK); health insurance (employee health insurance, private insurance, public hospitals-no insurance); educational level (incomplete primary school, complete primary school, incomplete secondary school, complete secondary school, incomplete tertiary school, complete tertiary school, incomplete university degree, complete university degree); current occupation (student, retired, employee, homemaker, self-employed, unemployed, informal work, NR/DK, other); “have you ever had sex in exchange for money, help, protection, goods or gifts?” (yes, no); and “have you ever had… Hepatitis B or C, syphilis or HPV?” (yes, no-for each STI). The questionnaire was self-administered and peer health promoters were helping and dissipating doubts during the process. TGW were tested as outlined in Fig 1.

TGW were allowed to repeat the test only if at least 3 months had passed from the previous one.

#### HIV rapid tests

A venous blood sample (25 ml) was drawn in 4 tubes (2 of them with EDTA) of which 5 ml were used to perform the detection of HIV infection using, simultaneously, the commercial RT Determine^™^ HIV-1/2 Ab and Determine^™^ HIV-1/2 Ag/Ab Combo (Alere Healthcare, SLU Barcelona, Spain) according to the manufacturer’s instructions *(*https://www.alere.com/en/home/products-services/infectious/hiv.html). The remaining 20 ml of blood was sent to the laboratory within 6 hours for plasma and serum processing.

#### Fourth-generation ELISA

After blood samples were centrifuged at 2500 rpm for 15 minutes at room temperature, the serum was separated and used for screening HIV antibodies with a 4^th^ generation ELISA assay (VIDAS HIV DUO Ultra, BioMérieux, Marcy l’Etoile, France).

#### Pooled viral load

For all participants whose results of the RT or the ELISA were negative, pools of 6 plasma samples were constructed, from which the detection of the viral RNA was conducted using the Abbott Real Time HIV-1 m2000rt method (limit of detection 40 copies/ml, Abbott Molecular Inc., Des Plaines, IL). If the pool was negative, no further tests were performed. The pools with positive results were analyzed individually to identify the positive sample(s) with the same molecular test.

#### Follow-up and confirmatory testing

In case of a positive result, by any diagnostic method used, a new viral load (VL) was used as a confirmatory method. CD4+ T cell count was also performed. In order to consider treatment initiation, HIV-positive individuals were linked to an Infectious Disease physician with a facilitated appointment to start antiretroviral therapy, including the cases detected by HIV plasma RNA.

#### Ethics statement

The institutional review board of a non-for-profit research organization (ethics committee Fundación Huésped FWAIORG0001557) approved this study (FH-20). All participants provided written informed consent. Participation was voluntary, and upon completion, participants received a $100 ARS reimbursement (approximately, $10 dollars at the moment of the study) for their time completing a social and behavioral questionnaire.

#### Statistical analysis

Given it was an exploratory study, a sample size was not estimated. Instead, a convenience sample was chosen to include every TGW who was willing to participate and get voluntarily tested during the study period.

Using both HIV testing methodologies, cases were codified as follows: 1) *positive*, cases of HIV infection by any test (RT, ELISA or VL) followed by confirmation with a new VL; 2) *AHI*, participants with an ELISA or RT Determine^™^ HIV-1/2 Ab negative, but a Determine^™^ HIV-1/2 Ag/Ab Combo or VL positive; 3) *non-infected*, individuals with negative results in all methods used.

Descriptive statistics were used to summarize the baseline characteristics of participants and incidence rates. Pearson’s chi-square test for categorical variables and the Wilcoxon rank sum test for continuous variables were performed to examine bivariable associations between independent variables of interest and HIV-positive results. All variables found to be associated with the outcome at p < 0.10 were then entered into a multivariable logistic regression model. Two-sided p-values as well as unadjusted and adjusted ORs (AOR) with 95% confidence intervals (CI) are reported. All analyses were performed with SPSS version 22.0 (IBM SPSS Statistics for Windows. Armonk, NY: IBM Corp., 2013).

## Results

### Characteristics of the sample

A total of 260 TGW were offered to participate and were tested through the aforementioned battery of HIV tests. No person refused to participate or was excluded. The median age was 27 years (IQR 23.0–36.7), 87.6% had no health coverage and 86% of the participants reported a history of sex work. Demographic characteristics of the sample are shown in Table 1.

**Table 1:** Demographic characteristics and bivariate logistic regression of the factors associated with HIV diagnosis among TGW in Argentina.

Characteristic		HIV diagnosis n (%)		Odds Ratio (95% CI)	*p-*value
	Total n = 260	Positive n = 53	Negative n = 207		
**Age, median (IQR)** ^b^	**27.5 (23.0–36.7)**	**28 (24.0–34.5)**	**27 (22.0–38.0)**	**0.99 (0.96–1.02)**	**0.853**
**Foreign Born** ^b^					
**yes**	**25 (11.0)**	**8 (17.8)**	**17 (9.3)**	**2.11 (0.84–5.26)**	**0.090**
**no**	**203 (89.0)**	**37 (82.2)**	**166 (90.7)**		
**Highest educational attainment** ^b^					
**High school or greater**	**91 (35.3)**	**17 (32.7)**	**74 (35.9)**	**0.86 (0.45–1.65)**	**0.396**
**Less than high school**	**167 (64.7)**	**35 (67.3)**	**132 (64.1)**		
**Health insurance** ^b^					
**yes**	**29 (12.4)**	**4 (8.9)**	**25 (13.2)**	**0.64 (0.21–1.94)**	**0.304**
**no**	**205 (87.6)**	**41 (91.1)**	**164 (86.8)**		
**History of sex work**^a^ ^b^					
**yes**	**215 (86.0)**	**42 (80.8)**	**173 (87.4)**	**0.60 (0.27–1.36)**	**0.159**
**no**	**35 (14.0)**	**10 (19.2)**	**25 (12.6)**		
**Current sex work**^b^					
**yes**	**148 (59.0)**	**33 (66.0)**	**115 (57.2)**	**1.45(0.75–2.77)**	**0.166**
**no**	**103 (41.0)**	**17 (34.0)**	**86 (42.8)**		
**Previous STI**^a^ ^b^ ^c^					
**yes**	**84 (32.3)**	**23 (43.4)**	**61 (29.5)**	**1.83 (0.98–3.41)**	**0.040**
**no**	**176 (67.7)**	**30 (56.6)**	**146 (70.5)**		
**HIV+ partner** ^a^ ^b^					
**yes**	**28 (12.9)**	**5 (12.8)**	**23 (12.9)**	**0.99 (0.35–2.79)**	**0.613**
**no**	**189 (87.1)**	**34 (87.2)**	**155 (87.1)**		
**Condom use at last sexual relation** ^b^					
**yes**	**159 (64.6)**	**26 (54.2)**	**133 (67.2)**	**0.57 (0.30–1.09)**	**0.065**
**no**	**87 (35.4)**	**22 (45.8)**	**65 (32.8)**		
**First HIV test** ^a^ ^b^					
**yes**	**47 (19.2)**	**19 (39.6)**	**27 (13.6)**	**4.17 (2.05–8.46)**	**0.000**
**no**	**198 (80.8)**	**29 (60.4)**	**172 (86.4)**		

**Abbreviations**: IQR, interquartile; STIs, sexually transmitted infections;

^a^ Denotes lifetime experience.

^b^ Among participants with a valid response to this question.

^c^ Syphilis, gonorrhea, human papilloma virus, hepatitis B and C.

### Comparison of different HIV testing methodologies

Fifty-one cases with positive Abs were documented with the RT, which represent a prevalence of 19.6%. All positive cases were confirmed with further serology and VL, therefore, no false positive case was found. The median CD4+ T cell count was 432 cells/μl (IQR: 219–667 cells/μl) but 23.2% had less than 200 CD4+ T cells. There were no discordant results between both RTs nor between RT and 4^th^ generation ELISA, therefore the correlation was 100%. The VL pools identified two additional positive samples (negative Ab and negative p24 by RT and 4^th^ generation ELISA but positive VL). The final analytic sample for positive cases consisted of 53 TGW.

During the study period, 25 TGW were re-tested on more than one occasion, with an average of 9 months (range 6–14 months) between tests. Three out these 25 cases seroconverted during this period. Considering these five cases (2 detected during AHI and 3 who seroconverted during follow up) the estimated incidence rate was 22.2.

### Factors associated with an HIV-positive diagnosis

In the multivariate analysis (Table 2), factors associated with a positive HIV result were *history of other STIs* and *not being tested for HIV previously*. The TGW tested for the first time had a greater than 4 times risk of being HIV positive compared with those that were tested previously.

**Table 2:** Multivariable logistic regression analysis of factors associated with HIV diagnosis among transgender women in Argentina.

Variable	Adjusted Odds Ratio (AOR)	95% Confidence Interval (CI)	p—value
**Foreign born**			
**(yes vs. no)**	**1.33**	**(0.38–4.60)**	**0.645**
**History of sex work**^a^			
**(yes vs. no)**	**0.80**	**(0.21–2.96)**	**0.739**
**Current sex work**^b^			
**(yes vs. no)**	**1.57**	**(0.64–3.86)**	**0.322**
**Previous STI**^a^			
**(yes vs. no)**	**3.36**	**(1.47–7.68)**	**0.004**
**Condom use last sexual relation**^b^			
**(yes vs. no)**	**0.43**	**(0.19–0.97)**	**0.044**
**First HIV test**^a^			
**(yes vs. no)**	**4.86**	**(2.03–11.66)**	**0.002**

^a^ Denotes lifetime experience.

^b^ Denotes current behaviour or activity.

### Feasibility of a multidisciplinary home-based HIV diagnosis intervention for TGW

Regarding feasibility, HIV testing through home-based intervention among TGW showed high uptake of testing, 100% of TGW who were offered the intervention agreed to be tested. The multidisciplinary approach was very well received by TGW, particularly the inclusion of peer health promoters. This research did not include an acceptability study, nevertheless the high uptake and the fact that 25 TGW asked to be re-tested on more than one occasion, shows a tendency towards acceptability.

## Discussion

Our study revealed a strong correlation between the HIV Ag/Ab RT and the 4^th^ generation ELISA tests, as well as the additional contribution of including VL pools to improve the HIV diagnosis rate. In terms of factors associated with HIV infection, not being tested for HIV previously and a self-reported history of STIs were related to positive results. Overall, HIV RT and VL pooling included in a multidisciplinary home-based testing strategy proved to be a feasible intervention for and HIV testing scale-up and diagnosis of AHI among TGW in Argentina.

### Comparison of different HIV testing methodologies

The performance of the two rapid assays (Determine^™^ HIV-1/2 Ab and Determine^™^ HIV-1/2 Ag/Ab Combo) and the 4^th^ generation ELISA VIDAS DUO was evaluated, showing similar sensitivity and specificity. There were no false positive cases and two false negative results were further identified with VL pooling. This finding supports the inclusion of molecular diagnosis techniques into the testing HIV algorithm for high-risk populations. Optimal HIV diagnostic algorithms should be easy, practical and with a low rate of false-positive results, as well as being affordable, mainly in low/middle-income countries. Although the inclusion of Ag/Ab assays in the algorithm allows reduction of the diagnostic window, they fail to identify participants in Fiebig stage I, a situation where early treatment could potentially provide additional benefits in regard to the reservoir’s size [20,21]. The above mentioned molecular techniques are usually expensive, so, alternative approaches such as pooling are proposed as a way to increase its cost-effectiveness [28].

In our study, the estimated that the HIV prevalence was 19.6% which is lower than the previously reported 34% in the country for TGW [2,3,1]. Still, these results showed that TGW continue to be one of the most affected key populations. Over the last few years the Gender Identity Law has promoted healthcare access in trans-sensitive settings and motivated new prevention and diagnostic strategies targeting TGW, which could possibly have had a positive impact on HIV prevalence [10–29]. Nevertheless, it was found that the incidence continues to be extremely high in this group.

The diagnosis of AHI is very important for various reasons: epidemiological (a period of higher rate of transmission), immunological (preservation of CD4 levels), scientific (the opportunity to study virological, immunological and genetic mechanisms) and therapeutic (modification of natural history) [30,31]. These results suggest that in Argentina, additional diagnostic strategies are needed with a particular focus on TGW, to increase diagnosis. With this purpose in mind, molecular tests should be incorporated in the testing algorithm for TGW.

In line with a study conducted in Uganda, which showed that home-based testing is more efficient than hospital-based strategies to reach individuals with higher CD4 counts, the median CD4+ T cell count of our domiciliary sample was relatively higher than the national average obtained in hospital-based samples [3–32]. Provider-initiated testing strategies (including home-based approaches) are needed to gain early access to key populations as part of the strategy to reduce the late presenters´ rate. Particularly when considering that despite having free-of-charge access to testing and ARV therapy in the country, AIDS-associated mortality rates have remained stable in recent years in Argentina [3].

### Factors associated with and HIV-positive diagnosis

Factors associated with HIV infection were not being tested for HIV previously and self-reported history of STIs, which emphasizes the importance of implementing provider-initiated HIV testing. According to the Center for Disease Control and Prevention´s (CDC) 2006 recommendations for HIV testing, all persons at higher risk for HIV infection should be screened at least annually [33]. Although the evidence was insufficient to recommend screening more frequently, based on our results [34] and the fact that three individuals out of the 25 who tested more than once, presented an HIV seroconversion, healthcare providers should consider offering tests more frequently, even every 3 months, when working with groups at higher risk for acquiring HIV, such as TGW with previous STIs and involved in sex work.

Of note, only 80% of the participants reported having been tested before, which highlights two issues: a) it reinforces the importance of increasing the frequency of testing in this population, and b) here is a group that does not access testing services and carries the highest burden of disease, with more than a 4 times higher risk of being HIV positive, according to our results, that could be reached with home-base strategies. The implementation of expanded testing programs, with different strategies, including home-base testing, self-testing, and other ways to facilitate access to individuals at risk and individuals that never tested before should be prioritized [35–36].

### Feasibility of a multidisciplinary home-based HIV diagnosis intervention for TGW

Home-based rapid testing has been evaluated as a highly acceptable and feasible intervention in community settings aimed at minority groups at high risk for HIV infection [15–37,38]. In this same line, our results show a high uptake in TGW and a tendency towards acceptability, identified by a series of indicators: 1) in the preparatory phase, the demand of home-based testing in a qualitative study [27], 2) the actual number of participants tested, 3) the fact that no one that had been offered to test refused to do it, and 4) the number of participants that requested to be re-tested.

Likewise, due to the particular characteristics of this population, the multidisciplinary approach was welcomed by TGW, particularly the inclusion of a peer health promoter among the members of the team. Similar to previous studies, peer health promoters facilitated access to venues and related more closely with TGW since they share background, culture, language and knowledge of the TGW community, and these should be included in every strategy targeted to this population [39,40,41]. Further, the participation of peer health promoters helped to administer the social and behavioral questionnaires by explaining technical vocabulary and responding to questions regarding the procedures.

Nevertheless, home-based HIV rapid testing strategies have raised some concerns, such as the potential for a confidentiality breach and for ineffective linkage to healthcare [42]. We faced similar concerns regarding confidentiality because of the high level of HIV intra-group stigma present among TGW in this setting, and the involvement of a person of the same community (i.e., a peer health promoter) [27]. However, based on our experience, having a multidisciplinary team properly trained and aware of the importance of confidentiality reduced the risk of breaking this ethical principle. Moreover, rather than interfering, having a TGW on the team has facilitated access to TGW venues and, for those individuals who got an HIV-positive result, the peer healthcare promoter has acted as a facilitator to engaging in care with health-care services by staying in contact after the intervention.

Additionally, it was pivotal to count upon with a private and quiet room to guarantee privacy and confidentiality, with some structural requirements such as avoiding high ceilings, low walls, doors that do not close or very small places. Besides the final results of the test, all participants left that room with the same amount of material, such as flyers and prevention method description. In the case of a positive result, this information, along with the medical appointment, was delivered on a small piece of paper to easily put away in a pocket or other safe place, and to avoid any possibility of unintentional disclosure. Therefore, the inclusion of peer health promoters, reduces the risk of breaches in confidentiality and facilitates linkage to healthcare. Additionally, in some settings where HIV stigma is higher, these concerns can be solved by involving a multidisciplinary team from a separate geographic location [42].

Multidisciplinary approaches have proven to increase engagement and retention in HIV care [43] but there is a lack of information, according to our knowledge, regarding the optimal composition of a multidisciplinary team for home-based HIV testing. Most home-based strategies for HIV tests only include individual/s trained specifically for such tasks such as community health workers, lay counsellors or nurses [44,45]. We had a positive experience with the team formed by infectious disease physicians, psychologists, laboratory technicians and peer health promoters. This particular composition of the team integrated counseling on STI prevention, allowed a better distribution of tasks and time, helped prevent and solve any problems or errors that may occur during the intervention, and answered different questions that TGW may have regarding HIV, STIs and other health issues, referring if necessary.

### Limitations

The present study has some limitations. First, the results may not necessarily be generalizable to all TGW in Argentina, especially those in rural areas, in particular, because the study recruitment was conducted in four large cities of Argentina. Second, as mentioned before, the study had a convenience sample. Future studies would benefit from expanding efforts to reach different sorts of venues and subgroups of the transgender population. Still, the final sample showed socio-demographic characteristics that are similar to nationwide studies with non-clinical samples [10].

Third, as the study relied on self-reported information, the answers could be shaped by low recollection of past events influencing, for example, not remembering the exact date of their last HIV test, or social desirability bias when filling out the social-behavioral questionnaire. This could have affected the analysis of the factors associated with the positive results, as these were based on the self-reported measurements. However, previous studies have used self-reported measures with good results [46]. Nonetheless, the presence of a peer health promoter, who is well-known and trusted in the TGW community, may have decreased the extent of social desirability bias and might have minimized the effect.

Finally, a home-based multidisciplinary strategy such as the one describe in this study might not be possible to reproduce in other settings due to lack of human resources, costs, etc. However, home-based multidisciplinary strategies are necessary to facilitate access to diagnosis, in particular for TGW, and other key populations that encounter multiple barriers to accessing the health care system, mainly due to the impact of stigma and discrimination [11,12]. The multidisciplinary team has the ability to integrate counseling on STI prevention, to explore risk behaviors and to address others health related issues [43] among TGW, and may facilitate healthcare access by helping to regain trust in healthcare workers specially among those who suffered from discrimination before [11*–*27]. Further studies should evaluate the best care team design in home-based strategies and analyze cost-effectiveness of these strategies.

## Conclusions

A multidisciplinary home-based HIV testing service among TGW is feasible and effective to detect cases of HIV infection, in particular among those that never were tested before. The testing algorithm should start with RT followed by molecular diagnosis, as the incidence in this group is high. The history of STIs and never having been tested for HIV were the factors associated with HIV-positive results and should determine efforts to reach this population.

## Supporting information

S1 File Social and behavioral questionnaire. Original in Spanish.
(PDF)

S2 File Social and behavioral questionnaire. English translation.
(PDF)

S3 File Database. (XLSX)
