# Supplementary material for: Home-based HIV testing: Using different strategies among transgender women in Argentina
Source: PLoS One. 2020 Mar 19;15(3):e0230429. doi: 10.1371/journal.pone.0230429 (PMC7081978; doi:10.1371/journal.pone.0230429)
Supplement: S1 File — Original in Spanish. (PDF) [file pone.0230429.s001.pdf]

Nº DE PROTOCOLO: FECHA:

Guía de entrevista de asesoramiento con test rápido de VIH

- **Edad:** ____________ **Género:** ○ Femenino ○ Masculino ○ Trans ○ Otro
- **¿En qué barrio vivís?** ______________________ **Nacionalidad:** ____________
- **¿Por qué querés hacerte el test de VIH?** (Podés marcar más de una)
  - ○ Me parece que puedo tener VIH
  - ○ Quiero estar seguro/a de que no tengo VIH
  - ○ Tuve una conducta de riesgo
  - ○ Me lo prescribieron para una práctica médica
  - ○ Me lo solicitaron en los exámenes prelaborales
  - ○ Otras: ______________________
- **¿Te hiciste el test alguna vez?** (indicá el año del análisis más reciente)
  - ○ No, es la primera vez ○ Sí, fue positivo (Año: ______) ○ Sí, fue negativo (Año: ______) ○ Sí, pero no retiré el resultado (Año: ______) ○ Ns/Nc
- **¿Querés recibir información de Fundación Huésped?**
  - ○ Sí email: ______________________
- **¿Con quién/es tenés relaciones sexuales?** (marcar todas las que correspondan)
  - ○ Hombres ○ Mujeres ○ Trans ○ No tengo relaciones ○ Prefiero no contestar
- **Parejas sexuales en el último mes** ______________________
- **Última relación sexual sin preservativo** ______________________
- **Síntomas en las últimas 2 semanas (ej: fiebre, ganglios aumentados, lesiones en la piel...)** ______________________
- **¿Utilizaste preservativo en tu última relación?** ○ Sí ○ No ○ Ns/Nc
- **¿Alguna vez tuviste una pareja con VIH?** ○ Sí ○ No ○ Ns/Nc
- **Cobertura de salud:**
  - ○ Obra Social — ¿Cuál?: ____________ Número: ____________
  - ○ Pre-paga — ¿Cuál?: ____________ Número: ____________
  - ○ Sistema Público — ¿Cuál es tu centro de referencia? ____________
- **Estudios cursados:**
  - ○ Primaria incompleta ○ Secundaria incompleta ○ Terciario incompleto ○ Universitario incompleto
  - ○ Primaria completa ○ Secundaria completa ○ Terciario completo ○ Universitario completo

Nº DE PROTOCOLO: FECHA:

Ocupación:

- Estudiante solamente
- Jubilado/a o pensionado/a
- Empleado/a en relación de dependencia
- Ama/o de casa
- Monotributista o Responsable Inscripto
- Otro: ________________________
- Desocupado/a
- Trabajo informal/Changas
- Ns/Nc

¿Cuál es tu oficio/profesión? ____

¿Recibís algún subsidio? No Sí ¿Cuál? ____________________________

Marcá con una X lo que corresponda

ALGUNA VEZ...	**SI**	**NO**
Recibiste sangre antes de 1990		
Usaste drogas en forma inyectable		
Tuviste sexo a cambio de dinero, ayuda, protección, bienes o regalos		

ALGUNA VEZ TUVISTE...	**SI**	**NO**
Fiebre prolongada sin causa (>7 días)		
Mononucleosis infecciosa		
Hepatitis viral de tipo B o C		
Diarrea por más de 3 meses		
Cáncer o tumores		
Herpes Zoster (culebrilla)		
Sífilis o gonorrea		
Cándida oral o vaginal		
Lesiones por virus papiloma (HPV) o verrugas genitales		

PARA SER COMPLETADO POR PERSONAL DE FUNDACIÓN HUÉSPED

Entrevistador/a: ____

Código del consultante:

Sexo (F-M)	1°y 2° letra del primer nombre	1°y 2° letra del primer apellido	Fecha de nacimiento (dd/mm/aaaa)

CONSENTIMIENTO INFORMADO DEL/LA CONSULTANTE

Ciudad de Buenos Aires, _______/_______/201___

Declaro en forma libre y voluntaria, con plena capacidad para ejercer mis derechos, que he sido suficientemente informada/o de la con¬venien¬cia de realizarme el test de tamizaje para la detección del VIH. Se me ha explicado en qué consiste la prueba, los beneficios del diagnóstico temprano para el cuidado de la salud y el alcance y significado de los resultados. Se me ha asegurado también la confiden¬cia¬lidad de los resultados y me han sido comunicados mis derechos al debido asesoramiento y la asistencia en caso de estar infectada/o por el VIH, en el marco de la legislación vigente. Asimismo, he sido informado que si la primera prueba fuera positiva pueden necesitarse muestras adicionales para confirmar la infección. Por lo expuesto, consiento expresamente que se me efectúe dicha prueba.

Firma: ____
