# Supplementary material for: Home-based HIV testing: Using different strategies among transgender women in Argentina
Source: PLoS One. 2020 Mar 19;15(3):e0230429. doi: 10.1371/journal.pone.0230429 (PMC7081978; doi:10.1371/journal.pone.0230429)
Supplement: S2 File — English translation. (PDF) [file pone.0230429.s002.pdf]

Case number: Date:

Interview guide for HIV rapid test counseling

Age: Gender: -Female -Male -Trans -Other

Neighborhood: Nationality:

Why do you want to have an HIV rapid test? (you can mark more than one)

- I think I might have HIV
- I want to be sure I don't have HIV
- I had a risk behavior
- It was prescribed for a medical intervention
- It was requested for pre-employment exams
- Other:..

Have you ever done an HIV test? (indicate the year of the most recent test)
-No, it's the first time -yes, it was positive (year:....) -yes, it was negative (year:....)
-Yes, but didn't return for the result (year) - NR/DK

Do you want to receive information about Fundación Huesped?
-Yes email:...

With whom do you have sexual relations?
-Men -Women -Trans -I don't have sexual relations -Prefer not to answer

Sexual partners in the last month (number):........................

Last sexual encounter without a condom (date):.....................

Symptoms in the last 2 weeks (e.g.: fever, swollen lymph nodes, skin lesions…):...................

Did you use a condom at your last sexual encounter? -yes -no -NR/DK

Have you ever had a sexual partner with HIV? -yes -no -NR/DK

Health insurance:
-Employee health insurance: Which one?......................Number:...................
-Private insurance: Which one?......................Number:..................
-Public hospitals (no insurance): Which one?......................Number:..................

Educational level:

-Incomplete primary school -Complete primary school -Incomplete secondary school -Complete secondary school -Incomplete tertiary school -Complete tertiary school -Incomplete university degree -Complete university degree

Do you receive social welfare benefit? -No -Yes -Which one?......................

Occupation:

-student -homemaker -unemployed

-retired or pensioner -self-employed -informal work
- employee -other:.............. -NR/DK

What is your occupation/profession?:..

Do you receive a subsidy? (*which)

Mark your response with an ”X”
Have you ever…?:

	SI	NO
Received a blood transfusion before 1990		
Used injectable drugs		
had sex in exchange of money, help, protection, goods or gifts		

Have you ever had…?:

	SI	NO
Persistent fever without a cause (>7 days)		
Infectious mononucleosis		
Hepatitis B or C		
Diarrhea for more than 3 months		
Cancer o tumors		
Herpes Zoster (shingles)		
Syphillis or gonorrhea		
Oral or vaginal candida		
HPV lesions or genital warts		

To be completed by Fundación Huésped staff

Interviewer:...
Identification code of the participant (National code for HIV test):

Sex (F or M)........First two letters of the first name…..…First two letters of last name………...
Date of birth

Informed consent of the participant for HIV test

Ciudad de Buenos Aires,........./………./201…

I hereby freely and voluntarily declare, with full capacity to exercise my rights, that I have been sufficiently informed about the convenience of doing a test for HIV screening. I have been told what the test consists of, the benefits of early diagnosis for taking care of health, and the scope and meaning of the results. I have also been assured of the confidentiality of the results and have been informed of my right to counseling and assistance in the event of having an HIV infection, within the framework of current legislation. I have also been informed that if the first test is positive, additional samples may be needed to confirm the infection. Therefore, I expressly consent to such a test.

Signature:..
